# Acquired phimosis after plastibell circumcision: a preventable consequence

**DOI:** 10.1308/003588412X13373405384774

**Published:** 2012-09

**Authors:** EA Kidger, N Haider, A Qazi

**Affiliations:** Leeds Teaching Hospitals NHS Trust,UK

**Keywords:** Plastibell, Circumcision, Complication, Acquired phimosis

## Abstract

**INTRODUCTION:**

The plastibell device is used successfully for religious and cultural circumcisions in the community. The aim of this article is to highlight the recognition and management of iatrogenic phimosis.

**METHODS:**

A retrospective study was performed of outcomes of plastibell circumcision in a community-based circumcision service provided by trained paediatric surgeons. The objective was to assess the complication of slipped plastibell rings and to ascertain the effectiveness of its management.

**RESULTS:**

A total of 5 patients with a slipped plastibell ring were indentified out of 560 plastibell circumcisions. Three patients presented with acquired phimosis. In two patients early diagnosis and management prevented any further complications and a second operation was avoided.

**CONCLUSIONS:**

Plastibell circumcision in the community is safe and effective. Detection of minor bleeding due to a slipped ring is important. Early management can avoid the risk of acquired phimosis due to cicatrix formation and can save parents of undue anxiety.

The plastibell device has been used in circumcision for over 50 years.^1^ It is an effective tool for religious and cultural circumcisions in the community^1,2^ in the UK and US. Despite its success, the overall complication rate after plastibell circumcision has been documented to be as high as 20%.^5^ However, most studies have found the overall complication rate to be around 7%.^3,4^ Complications include bleeding, infection, loss of penile skin, meatal stenosis, incomplete circumcision, proximal migration of the plastibell and an iatrogenic phimosis due to scarring.^1,5^ Many of these patients present initially to their local accident and emergency department. The aim of this article is to**highlight that early detection of the misalignment of the inner foreskin and outer layer can prevent the formation of iatrogenic phimosis.

## Methods

A retrospective analysis was performed of the outcome of slipped plastibell rings culminating in phimosis.

### Plastibell technique

An incision is made through the dorsal foreskin under local anaesthesia. The plastibell device is positioned over the glans and the foreskin pulled over. A ligature is then tied around the foreskin over the groove of the plastibell.

### Pathogenesis of acquired phimosis

The purpose of the ligature is to appose both edges of the skin until the foreskin cuts off and the layers are fused. Early bleeding can occur as a result of a loose ligature or a smaller sized plastibell, leading to slippage of the inner layer of the skin. When the plastibell falls off, the wound heals with a scar due to the two layers being wide apart. The outer layer cicatrises gradually and results in a phimotic scar within three weeks. Corrective surgery is always required, which may risk large amounts of penile shaft skin.

## Results

**Figure 1:**
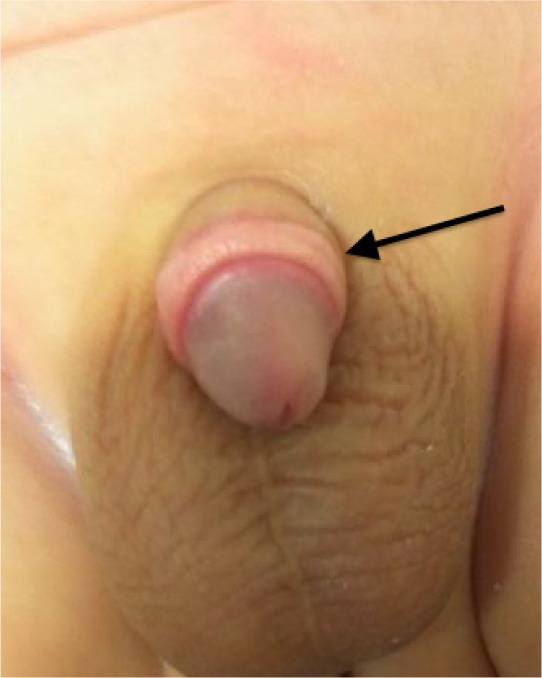
A nicely healed wound after regular retraction following a slipped plastibell ring and bleeding immediately after the circumcision

A total of 560 plastibell procedures were performed between April 2008 and September 2011. Five patients developed complications of a slipped ring and all were managed at different stages of this complication. Two patients presented within a few hours of circumcision with minor bleeding. One of these patients presented to our service directly and was therefore recognised at an early stage. The plastibell ring was removed and the wound formally sutured. The second patient did not inform our service but attended his local hospital for bleeding. He was discharged home once the bleeding had subsided and then presented to our service with early cicatrix formation within ten days of the initial procedure. On this occasion, regular retraction resolved the scar and the child avoided any further procedure (Fig 1).

**Figure 2:**
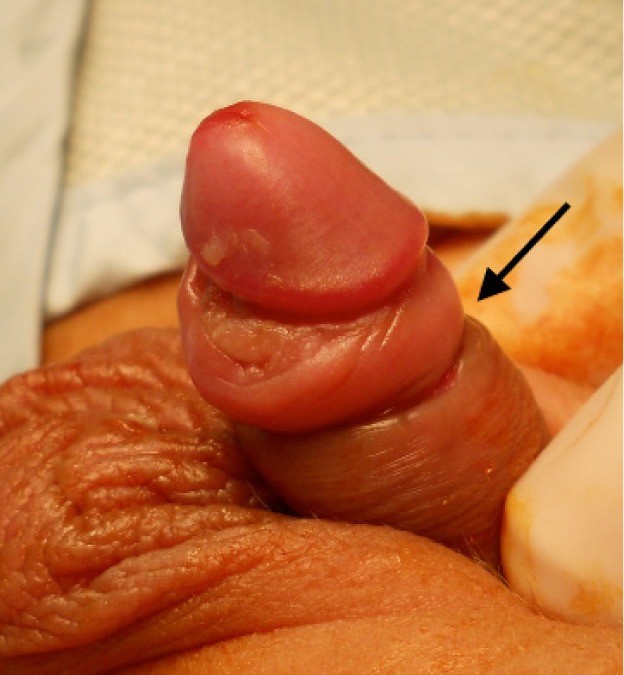
Acquired phimosis with tight scar

**Figure 3:**
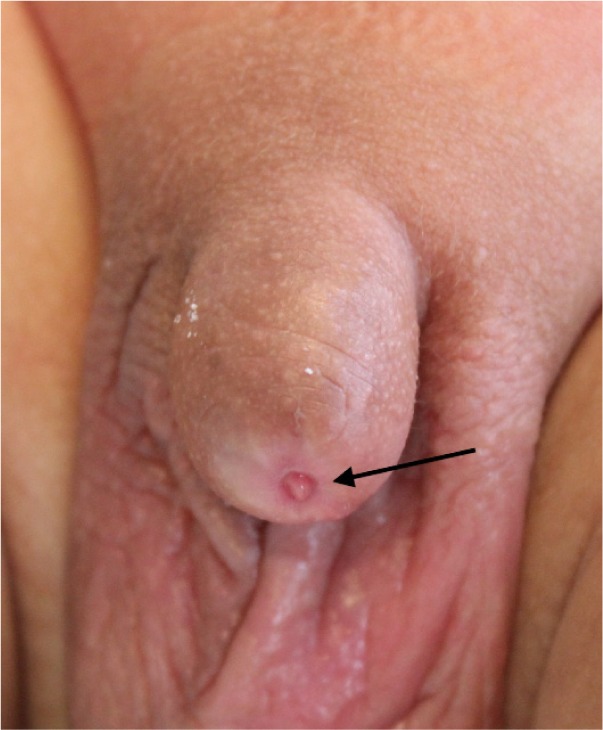
Tight ring of scar as seen after dorsal incision and retraction of scarred ring

**Figure 4:**
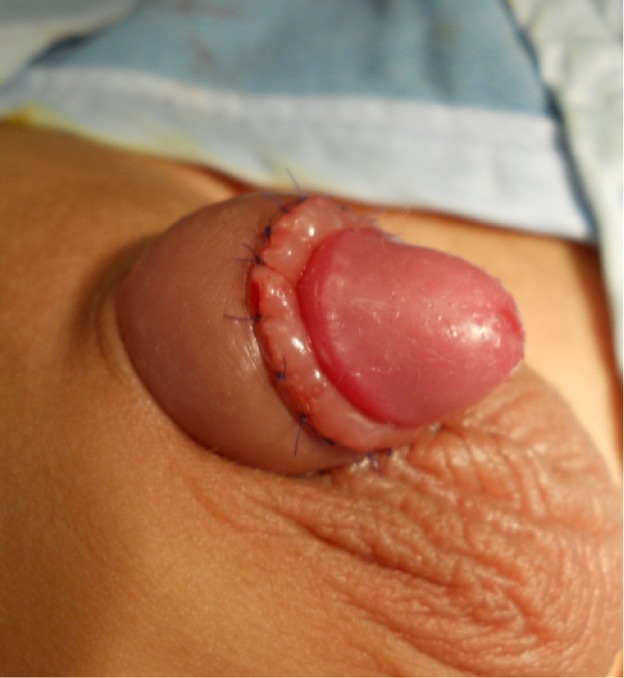
Revision of circumcision after excising the scar. Note the shorter length of available penile shaft skin.

Three patients had minor bleeding a few hours after the initial procedure but the parents did not attend hospital as the bleeding stopped spontaneously. These three patients then presented 1–3 months after the initial procedure. All of them had already developed phimosis and required revision to release the buried glans as an elective procedure after 4–6 months (Figs 2–4). All patients had a satisfactory final outcome of circumcision without any loss of penile shaft skin.

## Discussion

Plastibell circumcision has been shown to be a safe and effective method for use in the community. However, there are recognised complications, acquired phimosis being one. To prevent this complication, we suggest that practitioners should be competent in identifying minor bleeding as a result of a slipped ring. Slippage of the inner layer should be suspected if, following the circumcision, the glans appears to have retracted from the edge of the plastibell ring or if one can slide the tip of a mosquito clamp between the ring and glans deeper than the depth of the glans. The most common cause of bleeding is a torn frenular artery, which can be identified easily by the presence of rapid bleeding rather than minor ooze and the fact that it does not usually stop spontaneously. This can cause severe haemorrhage in a neonate and may even require urgent resuscitation and transfusion if presenting late.

Depending on the time of identification of the slipped ring, different management pathways can be used. If the plastibell is still in place, it should be removed and the wound formally sutured by a paediatric surgeon/urologist. If the plastibell has fallen off, regular retraction of the outer layer should be performed immediately and regularly. If this is not recognised and a cicatrix has developed, corrective surgery will be invariably required. It should be delayed for at least three months to reduce bleeding due to inflammation in the early wound healing phase.

## Conclusions

The use of the plastibell ring for circumcision is effective and it can be used safely in the community. Several complications are recognised, however, one being the formation of a cicatrix due to misalignment of the inner foreskin and outer layer. Identification of such misalignment at an early stage can alleviate this problem and it can then be managed with minimal surgical intervention.
